# Doping and Defect Engineering of CuO for Enhanced Performance in Si Heterojunction Solar Cells

**DOI:** 10.1021/acsomega.5c05088

**Published:** 2025-09-03

**Authors:** Adithya Prakash, Saikat Chattopadhyay, MG Mahesha

**Affiliations:** 1 Department of Physics, Manipal Institute of Technology, 76793 Manipal Academy of Higher Education, Manipal 576104, India; 2 Department of Physics, School of Physical and Biological Sciences, 385092 Manipal University Jaipur, Jaipur, Rajasthan 303007, India

## Abstract

CuO thin films are attractive candidates for solar cell absorber layers due to their natural abundance and suitable band gap. This study investigates the potential of pristine and doped (Zn, Mg, and Al) CuO thin films in Si-based heterojunction solar cells through both experimental fabrication and SCAPS-1D simulations under AM 1.5 illumination. Simulations revealed that doping significantly enhanced the solar cell efficiency, increasing it from 5% for pure CuO to over 24% in doped samples, approaching theoretical predictions. However, introducing parasitic resistance reduced the efficiency to 3.99% for pure CuO and 5.77% for Al-doped CuO. Additionally, oxygen defects were found to influence device performance, with higher efficiency observed when interstitial oxygen (*O*_*i*_) defects dominated over oxygen vacancies (*V*_*o*_). Experimental results confirmed the photovoltaic behavior in all fabricated devices. However, the efficiency of the fabricated device was very low compared with the simulated results for pristine and Zn- and Mg-doped CuO-based devices. Notably, Al-doped CuO exhibited a substantial increase in efficiency from 0.42% in pure CuO to 6.52%. The findings demonstrate that controlled doping and defect engineering in the absorber layer play vital roles in enhancing the performance of Si/CuO heterojunction solar cells. This approach holds promise for developing cost-effective and efficient photovoltaic technologies.

## Graphical abstract

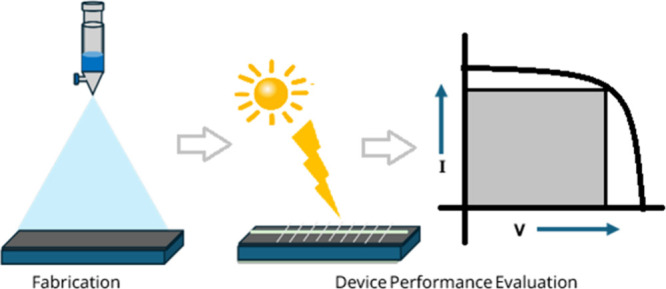

## 1 Introduction

The progress of current solar cells hinges significantly on their scalability and cost-effective manufacturing processes. This emphasizes the importance of the use of metal oxide thin films such as CuO in addition to simple and inexpensive synthesis methods such as spray pyrolysis for fabrication of solar cells. Among the oxides of copper, even though CuO has a narrower band gap that makes it a better fit for solar spectrum compared to Cu_2_O, there has been limited research on CuO solar cells until recently. The theoretical conversion efficiency of a homojunction CuO solar cell with the band gap of CuO being 1.4 eV is predicted to be 33%.^1^ However, due to the difficulty in preparing n-type CuO thin films, homojunction CuO solar cells are still a challenge. This has changed the scientific interest toward CuO-based heterostructures.

There are basically two types of CuO heterostructures reported so far: Si-based and FTO or ITO-based solar cells. The basic structure of a Si-based solar cell involves the deposition of p-type CuO thin films on an n-type Si substrate. In FTO/ITO-based solar cells, in addition to the CuO absorber layer, there is a window layer. The most widely used window layer used for CuO solar cells is ZnO. There are also reports in which TiO_2_ is used as the window layer. Among these heterostructures, FTO/ITO-based heterostructures are widely studied, and there are few available reports on Si-based heterostructures. In addition to these, several numerical simulation studies on CuO heterostructures have also been reported in the past few years.

Several parameters influence the performance of solar cells, including the thickness of absorber and buffer layers as well as the type of buffer layer employed. Numerical simulations provide insights into how these factors affect the properties of CuO solar cells.^2^ According to research by Chowdhury,^2^ thin absorber layers can reduce solar cell efficiency due to increased charge carrier recombination. The thickness of the buffer layer, while generally having a minimal impact on efficiency, can also contribute to charge carrier recombination when excessively thick. Among various buffer layers studied, simulations show that WS_2_ resulted in the highest efficiency at 19.6%.^2^ Additionally, operating temperature significantly affects CuO solar cell performance; higher temperatures decrease charge carrier concentration and increase recombination rates, thereby reducing overall solar cell efficiency.^2^

The incorporation of electron and hole transport layers has notably improved the conversion efficiency of CuO solar cells. A remarkable efficiency of 29.40% was achieved with FTO/SnS_2_/CuO/ZnTe heterostructures, where SnS_2_ functions as the electron transport layer and ZnTe serves as the hole transport layer.^3^ Similarly, research into employing V_2_O_5_ as the rear surface field layer demonstrated an enhanced efficiency in ITO/ZnO/CuO solar cells. The theoretically found efficiency was 2.05%.^4^ Benaissa modeled FTO/TiO_2_/CuO heterostructures and predicted a theoretical efficiency of 13.8%.^5^ Majority of these numerical studies predicted high efficiency for CuO solar cells, indicating their suitability in PV industry.

The experimental studies on CuO solar cells so far have reported very low efficiency compared to that of the numerical models. Most of these reported experimental studies on CuO/Si solar cells are based on the sputtering technique. Gao *et al.* have fabricated CuO/Si heterostructures with a conversion efficiency of 0.41%.^6^ The interface defects and the high series resistance of CuO layers resulted in a low value of FF and *V*_oc_. Masudy-Panah fabricated CuO heterostructures on Si substrates by RF sputtering.^7^ They also observed an amorphous oxide layer between CuO and Si. It was found that the efficiency of the devices fabricated depended on the quality of the thickness of this oxide interface layer. They observed an improvement in efficiency to 0.36% when the interface layer was thin. When a N-doped CuO layer was introduced between the CuO and top contact layer, the efficiency enhanced to 1%.

Solar cell efficiency can be enhanced by utilizing doped CuO as the absorber layer, where dopants increase the carrier concentration and improve conductivity. This improvement reduces resistive losses and boosts the overall efficiency of Cu–O-based solar cells. Numerous studies have explored the use of doped CuO in solar cell applications. For example, incorporating small amounts of titanium dopants has been found to significantly enhance the conductivity of CuO films, thereby increasing their efficiency.^8^ However, a high concentration of doping can result in poor performance as well. N-doping reduced the series resistance and thereby improved the FF, *J*_sc_, and η.^9^

The potential of spray deposited CuO and Zn, Mg, and Al-doped CuO thin films for PV applications has been discussed in detail in our previous works.^10–13^ We observed that spray pyrolysis is one of the easiest methods for obtaining quality CuO thin films that are suitable for device applications. The quality of the deposited films was significantly influenced by the deposition conditions.^10^ Zn, Mg, and Al doping significantly improved the electrical properties of CuO thin films. Doping enhanced the *O_i_* defects in CuO thin films, which resulted in the improvement of the electrical properties.^11–13^

To assess the effectiveness of spray-deposited pure Zn, Mg, and Al-doped CuO as absorber layers, it is imperative to study the photovoltaic properties of heterostructures based on these films. In this study, the performance evaluation of Si-based CuO solar cells was done using both experimental and simulation routes. For this purpose, n-type Si was heteropartnered with pristine and doped CuO layers. The simulation studies were carried out using SCAPS software with solar AM 1.5 illumination. In-depth investigations have explored the impact of thickness, doping, and intrinsic defects of the absorber layer on the fundamental parameters of Si/CuO solar cells. Furthermore, the devices were fabricated via a spray pyrolysis technique and analyzed by using suitable characterization techniques.

## 2 Methodology

### 2.1 Numerical Simulation

To assess the potential of the prepared thin films for use as absorber layers in solar cells, simulations were conducted on solar cells incorporating these films by using SCAPS-1D software. The solar cell optical and electrical characteristics can be modeled and simulated using SCAPS (solar cell capacitance simulator) simulation software. It serves as a robust tool for exploring the performance of solar cells under diverse operational conditions and design parameters. This simulation software package was designed by Burgelman and Nollet^14,15^ for the real-time simulation of the electrical characteristics of heterojunction solar cells. 1D differential equations that govern the steady state charge carrier conduction mechanism are solved in SCAPS-1D software. This simulation tool aids in comprehending the fundamental principles of solar cells and the critical factors influencing their performance.

The output current in a solar cell is contributed by the drift and diffusion of electron density *n* and hole density *h*. The electron and hole current densities, *J*_*n*_ and *J*_*p*_, are given by^16^

$$J_{n}=-\frac{\mu_{n}n}{q}\frac{\partial E_{F_{n}}}{\partial x}$$ 1

$$J_{p}=\frac{\mu_{p}p}{q}\frac{\partial E_{F_{p}}}{\partial x}$$ 2

where μ_*n*_ and μ_*p*_ are the mobility of electrons and holes and *E*_*F*_n__ and *E*_*F*_p__ are electron and hole Fermi levels. Also, the electron and hole continuity equations are^17^

$$\frac{\partial n}{\partial t}=\frac{1}{e}\frac{\partial J_{n}}{\partial x}+G-R$$ 3

$$\frac{\partial p}{\partial t}=\frac{1}{e}\frac{\partial J_{p}}{\partial x}+G-R$$ 4

The electric field *E* can be calculated from Poisson’s equations as^17^

$$\frac{\partial^{2}\psi }{{\partial x}^{2}}+\frac{q}{\varepsilon }\left[p\left(x\right)-n\left(x\right)+N_{D}-N_{A}\right]=0$$ 5

So that $E=\frac{\partial \psi }{\partial x}$ , where E is the permittivity, *N*_*D*_ and *N*_*A*_ are the density of donors and acceptors, *R* is the recombination rate and *G* is the generation rate, and *t* is the time. The Poisson and continuity equations provide a set of coupled differential equations (ψ, *n*, *p*) or (ψ, *E*_*F*_n__, *E*_*F*_p__) with correct boundary conditions at interfaces and contacts.^16^ SCAPS-1D computes the steady-state and small-signal solutions for this system numerically. Under standard conditions (a cell temperature of 25 °C and radiation of 1000 W/m^2^ with AM spectrum 1.5), it reproduces the performance of the cell.

#### Device Structure

Figure 1(a) depicts the schematic diagram of the heterojunction solar cell utilized for simulation. The heterostructure consists of n-Si, which is used as the substrate, and a p-CuO thin film as the absorber layer. A total of four devices were considered for the simulations, the details of which are given in Table 1. The parameters of different layers used for simulations of CuO solar cells are given in Table 2. The Si parameters are taken from the SCAPS library whereas CuO layer parameters were taken from Chowdhury.^2^ The material properties of the n-Si layer remained the same throughout the simulations. To align the simulated system with the experimental setup, the optical parameters of the CuO absorber layer were adopted from the experimental findings.

**Figure 1:**
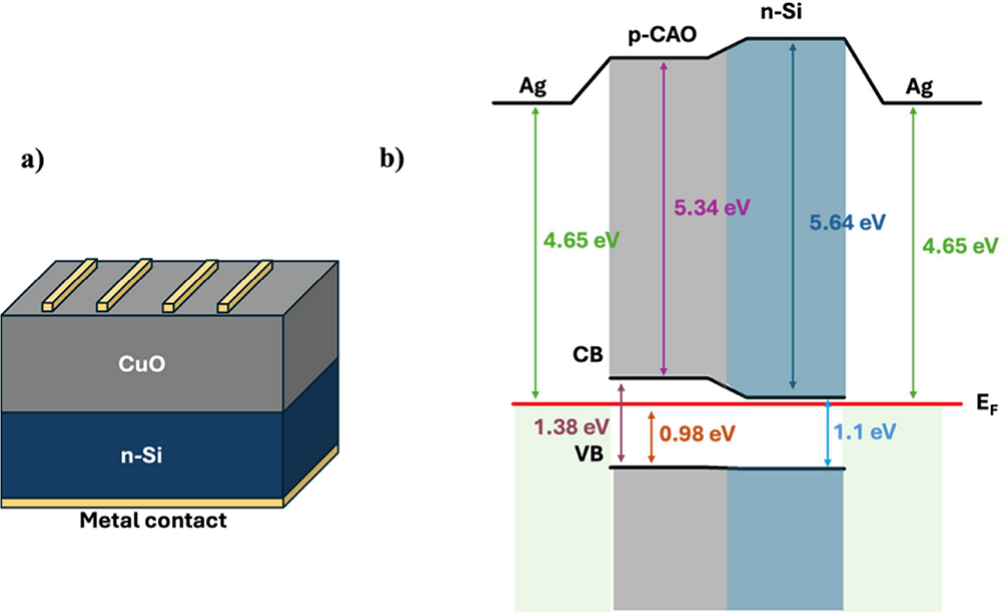
Schematic diagram of (a) device structure and (b) band diagram of the CAO solar cell.

**Table 1:** Details of Devices Used for Simulations

**device**	**heterostructure**	**absorber layer**	**band gap of absorber layer (eV)**
**CO**	n-Si/p- CuO	pure CuO	1.50
**CZO**	n-Si/p-CZO 6	6 at. % doped CuO	1.54
**CMO**	n-Si/p-CMO 10	10 at. % doped CuO	1.57
**CAO**	n-Si/p- CAO 1	1 at. % doped CuO	1.38

**Table 2:** Material Parameters Used for Device Simulation

**material parameters**	**n-Si**	**p-CuO**
**band gap (eV)**	1.12	experimental
**electron affinity (eV)**	4.050	4.070
**dielectric permittivity**	11.9	18.1
**CB effective density of states (1/cm** ^**3**^ **)**	2.80 × 10^19^	2.20 × 10^19^
**VB effective density of states (1/cm** ^**3**^ **)**	1.04 × 10^19^	5.50 × 10^20^
**electron thermal velocity**(cm/s)	2.30 × 10^7^	1.0 × 10^7^
**hole thermal velocity**(cm/s)	1.65 × 10^7^	1.0 × 10^7^
**electron mobility**(cm^2^/(V s))	1500	100
**hole mobility**(cm^2^/(V s))	450	0.1
**donor density (1/cm** ^**3**^ **)**	1.0 × 10^18^	0
**acceptor density (1/cm** ^**3**^ **)**	1.0 × 10^10^	1.0 × 10^16^

### 2.3 Experimental Details

#### Device Fabrication

Spray pyrolysis was used for the fabrication of CuO heterojunction solar cells; 0.25 M concentrated precursors of undoped and doped CuO absorber layers were deposited on n-Si substrates at a temperature of 450 °C. Precursor solutions were prepared by dissolving a suitable amount of chloride salts of Cu and Zn, Mg, and Al (for doping). After the deposition of the absorber layer, top and bottom Ag metal contacts were made using the PVD method. Figure 2 illustrates the device fabrication process. Figure 3(a) depicts the schematic model of undoped Si/CuO solar cells. Pure and 6 at. % Zn, 10 at. % Mg, and 1 at. % Al doped CuO absorber layers were deposited on Si forming four different device systems, which were named CO, CZO, CMO, and CAO, respectively. The optimization of deposition paraments and dopant levels is reported in our previous publications.^10–13^

**Figure 2:**
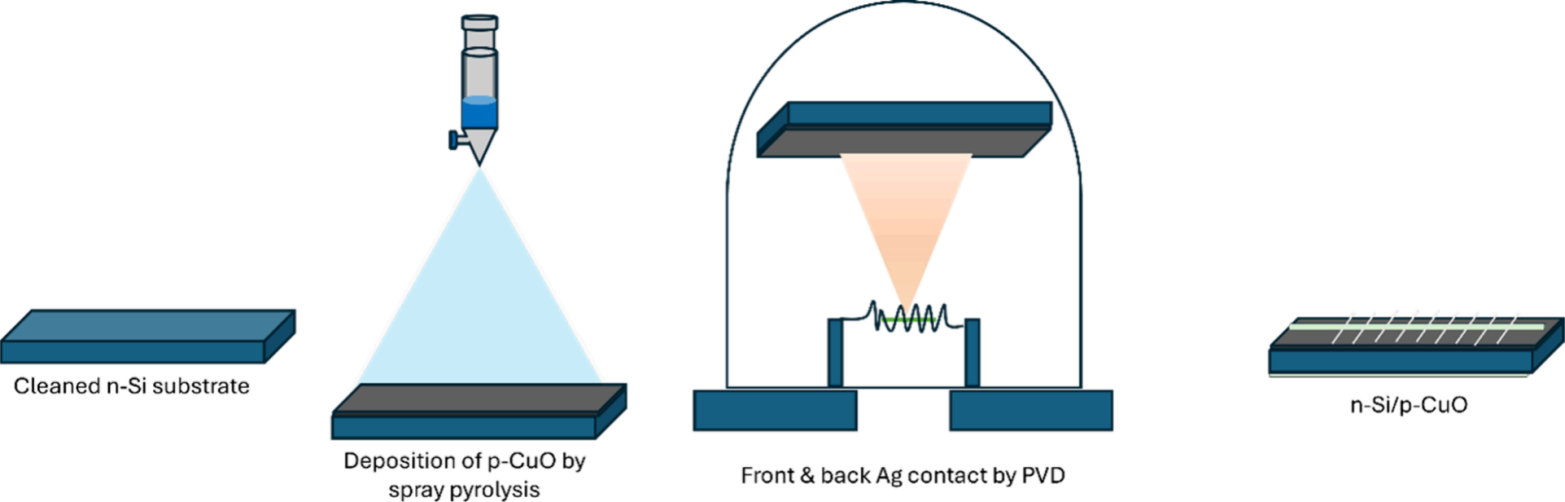
Schematic representation of the device fabrication process.

**Figure 3:**
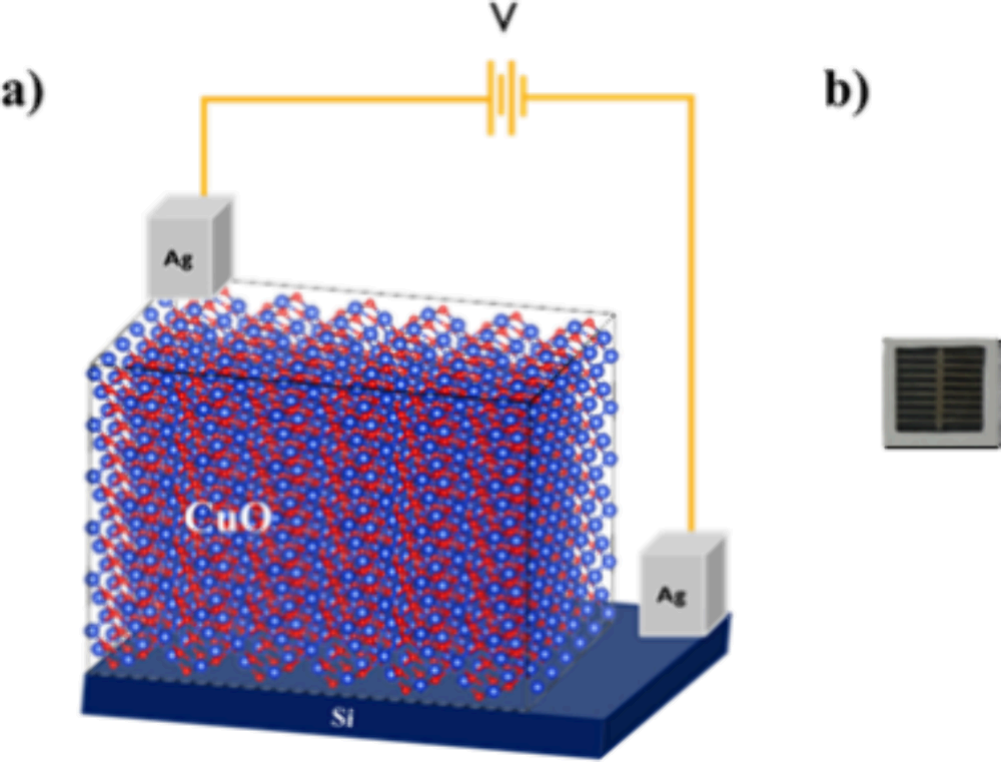
(a) Schematic model of the fabricated Si/CuO solar cell and (b) photograph of the fabricated device.

#### Device Characterizations

Structural properties of the devices were studied by using GI-XRD. The surface morphology of the devices was examined by using Zeiss SEM EV0 15. The reflectance spectra of the devices fabricated were recorded using a SHIMADZU UV 3600 spectrophotometer. Room-temperature Raman spectra of the films in the study were acquired using a LabRAM HR Horiba France model with a Nd:YAG laser of 532 nm as the excitation source. The behavior and efficiency of a solar cell in converting sunlight into electricity are evaluated through current–voltage (*I*–*V*) analysis using the Keithley 2450 SourceMeter. The UPS spectra of the devices were recorded at 300 K using an Omicron energy analyzer (Model no: EA 125, Germany). *I*–*V* characteristics of the fabricated devices were evaluated under both dark and illumination conditions. A Newport 67005 Xenon arc lamp powered by a Newport power supply 69907 was used as the light source for device characterization under illumination. The lamp is equipped with an Air Mass (AM) filter 1.5 for simulating the terrestrial solar spectrum.

## 3 Results and Discussion

### 3.1 Simulations of the Devices Using SCAPS

#### 3.1.1 Optimization of Thickness of the Absorber Layer

The illumination of a solar cell generates e-h pairs, the generation and separation of which are critically influenced by the absorber layer thickness. The impact of absorber layer thickness on the fundamental solar cell parameters was investigated through simulations of all modeled solar cells, as depicted in Figure 4. The simulations covered a range of absorber layer thicknesses from 0.5 to 20 μm, all conducted under standard conditions at a temperature of 300 K.

**Figure 4:**
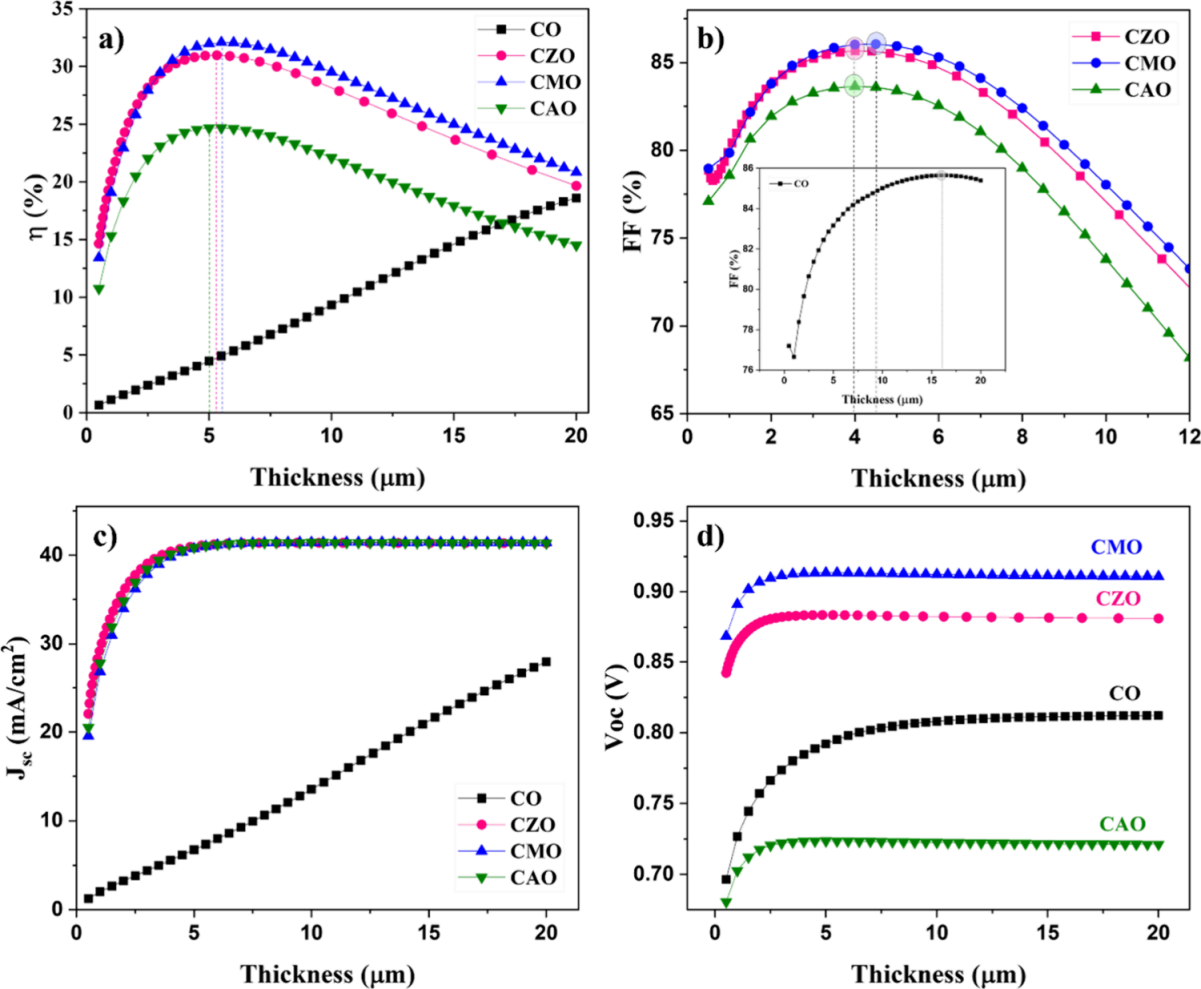
Dependence of solar cell parameters (a) efficiency, (b) fill factor, (c) short circuit current density, and (d) open circuit voltage on the absorber layer thickness.

Both FF and efficiency of the doped solar cells increased up to a certain value and then decreased with thickness. The maximum efficiency and FF were obtained at 5 μm for CZO and CAO and at 5.5 μm for CMO. On the other hand, for the CO sample, the efficiency and *J*_sc_ increased continuously until the maximum thickness considered for the calculations. However, FF was maximum at 16 μm, after which it decreased. *V*_oc_ of all the samples and *J*_sc_ of the doped samples showed a similar trend in the variation with the thickness. *J*_sc_ and *V*_oc_ of doped samples increased up to 4 μm thickness and remained the same with a further increase in thickness. However, the maximum value of *V*_oc_ of the CO was observed at a much higher thickness of about 16 μm.

Absorber layer thickness of solar cells greatly influences two key parameters of the solar cell: *J*_sc_ and *V*_oc_. A thicker absorber layer can capture more sunlight and hence generate a greater number of charge carriers, leading to an enhancement in *J*_sc_ and *V*_oc_. This explains the observed increase in *J*_sc_ and *V*_oc_ values up to an optimum thickness. At the optimum thickness, due to the built-in electric field, the depletion layer extends throughout the absorber layer that facilitates the extraction of maximum charge carriers. However, a further increase in the thickness decreases the depletion width and quasi neutral regions are observed at regions away from the junction.^18^ While a thicker absorber layer can generate a higher number of charge carriers, many of these carriers produced in the quasi-neutral region may not effectively cross the junction and are prone to recombination. Therefore, only a nominal increase in the charge carriers can be observed after the optimum thickness leading to a constant value of *J*_sc_ and V_oc._

The observed variations in FF and efficiency can also be explained by recombination losses. As the absorber layer thickness increases, the electric field strength across the junction also rises and peaks at an optimal thickness. This optimal thickness is characterized by a strong electric field that ensures that the absorber layer is thinner than the diffusion length of charge carriers, facilitating efficient charge carrier extraction. Hence, all the generated charge carriers travel across the junction without any recombination, resulting in maximum values of efficiency and FF at this thickness. Beyond this optimal thickness, the diffusion length of charge carriers becomes shorter than the absorber layer thickness. This leads to an increased recombination of the excess charge carriers generated, thereby reducing both the efficiency and the FF. In addition to the recombination losses, the resistive losses can also contribute to the decrease in the FF at higher thickness. As the thickness increases, series resistance also increases resulting in decreased FF.^19^

While the optimum thickness of the CO sample is 16 μm, the optimum thickness was reduced to one-fourth of the value after doping. This indicates that doping CuO with a suitable dopant can lead to a higher efficiency and improved performance even with thinner absorber layers. This is significant for lowering the manufacturing cost of Cu–O-based solar cells. Additionally, thinner absorber layers contribute to reduced resistive losses, further enhancing the overall cell performance. Next levels of simulation study were carried out by setting the absorber layer thickness to the optimum values obtained here.

#### 3.1.2 Influence of Doping

Figure 5 illustrates how doping influences the *J*–*V* characteristics of Cu–O-based solar cells. A significant improvement in *J*_sc_ is observed after doping. *J*_sc_ increased by more than five folds from 7.12 to 39.14 mA/cm^2^ for CZO, 40.99 mA/cm^2^ for CMO, and 40.88 mA/cm^2^ for CAO samples. *V*_oc_ showed a slight increase in Zn and Mg-doped samples, whereas it decreased in the case of the CAO sample. The devices exhibited a high FF of approximately 85%, indicating a significant enhancement in solar cell efficiency from 5.10% in the CO sample to an impressive 30.96% in the CZO sample. The CMO sample also showed a very high efficiency of 32% whereas the CAO sample showed an efficiency of 24.67%. The achieved efficiencies after doping approach the theoretical conversion efficiency of 33%, highlighting doping as a highly effective method for enhancing the efficiency of CuO-based solar cells.

**Figure 5:**
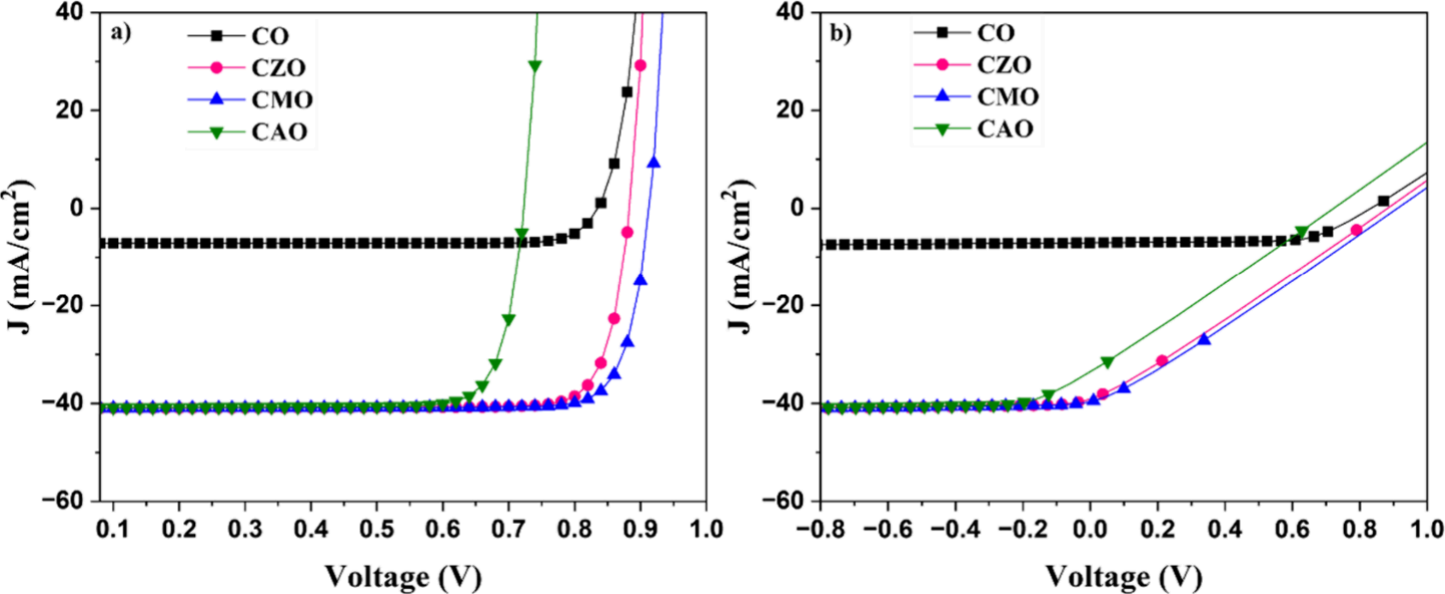
*J*–*V* characteristics of simulated solar cells (a) without and (b) with *R*_s_ and *R*_sh_.

The considerable enhancement in *J*_sc_ after doping can be attributed to the enhanced optical properties. Doping enhanced the light absorbance of the films, leading to the generation of a greater number of electron hole pairs, thereby increasing the *J*_sc_. The highest value of *V*_oc_ was observed for CMO followed by CZO, CO, and then CAO. The variation in the *V*_oc_ is associated with the *E*_g_ of the absorber layer. When incident photons carry energy greater than the band gap of the absorber layer, they are absorbed by the solar cell, causing electrons to transition from the VB to CB. This increases the potential energy of the electron by *E*_g_ = *qV*. If losses within the solar cell are negligible, *V* = −*E*_g_/*q* ^20^ is the maximum voltage obtained. Hence, *V*_oc_ increases with an increase in *E*_g_.

QE analysis provides insights into how solar cells perform under varying incident light wavelengths. Solar cells absorb different wavelengths at different depths. Blue light of low wavelength is absorbed at the surface, whereas red light is absorbed at a few 100 μm.

Figure 6 illustrates the wavelength-dependent absorption characteristics of simulated solar cells. The QE of all the samples is zero for the lower wavelength of light. The CO device showed a QE of nearly 20% for the wavelengths above 800 nm. A significant enhancement in the QE for wavelengths above 800 nm can be observed for all the doped samples. A high QE at larger wavelengths indicate the good quality of the absorber material because of the high diffusion length, i.e., the charge carriers generated at the rear side can successfully reach the junction without the recombination explaining the enhancement in the performance of doped solar cells.

**Figure 6:**
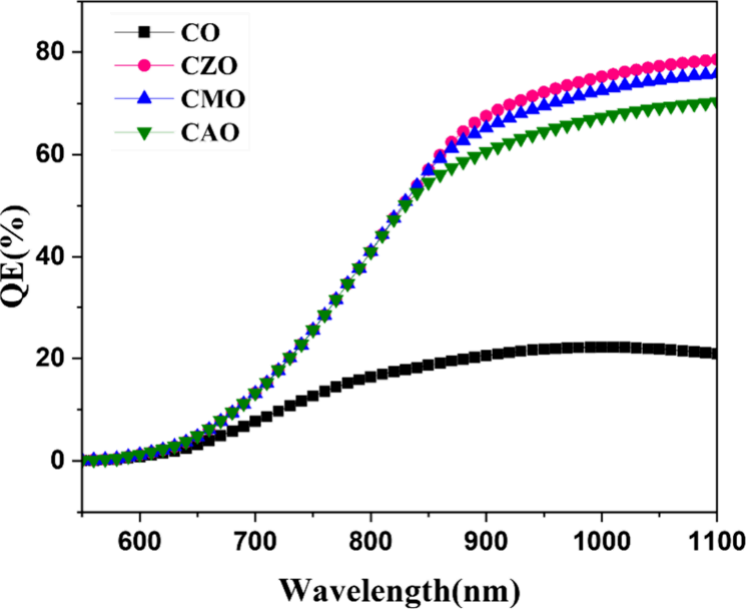
QE response of simulated solar cells.

In a practical scenario, solar cells experience several factors that diminish their performance, with losses from parasitic resistances being particularly significant. These resistances, which cannot be entirely avoided, exert a direct impact on the FF of the solar cell, thereby influencing its overall efficiency. Parasitic resistances manifest in two forms: series resistance, which affects the flow of current within the cell, and shunt resistance, which provides unintended paths for current to bypass the cell’s active region. The series resistance is due to the various components that come along the path of the current, and shunt resistance is due to the leakage across the junction. Ideally, the series resistance should be 0 and the shunt resistance should be ∞. However, it is not practically possible. To assess how series and shunt resistances impact solar cell performance, we simulated *J*–*V* characteristics. The simulations were configured with an *R*_s_ of 20 Ω and *R*_sh_ of 20 kΩ.

Figure 5(b) depicts the *J*–*V* characteristics derived from the simulations. The presence of parasitic resistances has noticeably modified the rectangular shape of the *J*–*V* curve, underscoring their impact on the FF of the devices. There has been a drastic reduction in the FF from 85 to nearly 67% in CO and around 25% in doped devices. The efficiency of the solar cells was correspondingly affected by the decline in FF. However, all of the doped devices showed a significant improvement in efficiency. The CMO and CZO devices showed an efficiency of 9.3% and CAO had an efficiency of 5.8%. On the other hand, the *V*_oc_ and *J*_sc_ values were unaffected. This indicates that the parasitic resistances have a negligible influence on these values. Table 3 presents the simulated characteristics of the devices.

**Table 3:** Device Parameters of the Simulated Devices for Different Conditions

**device**	**device parameters**	**without *R***_**s**_ **& *R***_**sh**_	**with** ***R***_**s**_ **= 20 Ω** **& *R***_**sh**_ **= 2 kΩ**	**with defects** *O*_*i*_ **= 1 × 10** ^**14**^ **cm** ^**–3**^ **&** *V*_*o*_ **= 1 × 10** ^**13**^ **cm** ^**–3**^
**CO**	*V*_**oc**_ **(V)**	0.84	0.84	0.83
	*J*_**sc**_(mA/cm^2^)	7.12	7.04	6.30
	**FF (%)**	85.64	67.03	66.86
	**η (%)**	5.10	3.99	3.48
**CZO**	*V*_**oc**_ **(V)**	0.88	0.88	0.88
	*J*_**sc**_(mA/cm^2^)	40.99	39.14	38.79
	**FF (%)**	85.46	26.78	27.36
	**η (%)**	30.96	9.26	9.36
**CMO**	*V*_**oc**_ **(V)**	0.91	0.91	0.91
	*J*_**sc**_ (mA/cm^2^)	40.98	39.67	39.30
	**FF (%)**	85.69	25.55	26.84
	**η (%)**	32.08	9.27	9.63
**CAO**	*V*_**oc**_ **(V)**	0.72	0.72	0.72
	*J*_**sc**_(mA/cm^2^)	40.88	33.64	33.25
	**FF (%)**	83.40	23.71	25.24
	**η (%)**	24.67	5.77	6.06

#### Influence of Oxygen Related Defects

The characteristics of CuO thin films are significantly influenced by intrinsic defects. These defects can exist at various levels within the bandgap of the semiconductor, either deep or shallow. Deep defects typically function as traps, causing e-h recombination and thereby diminishing the overall efficiency of solar cells. However, shallow defects, where the defect levels lie closer to the VB or CB, need not necessarily act as recombination centers. Among the different intrinsic defects, oxygen defects, namely *O*_*i*_ and *V*_*o*_, can be easily introduced and controlled in spray deposited thin films. *O*_*i*_ defects function as acceptors and can positively influence the p-type conductivity in CuO thin films whereas *V*_*o*_ defects act as a donor type and can adversely impact the conductivity. Both *O*_*i*_ and *V*_*o*_ defects can create either deep or shallow energy levels within the forbidden energy gap.

In order to investigate how acceptor defects influence CuO solar cell performance, a shallow acceptor defect level was introduced at 0.56 eV^21^ and *O*_*i*_ defect concentration was varied from 0 to 1 × 10^18^ cm^–3^. Figure 7 shows the variation in the device characteristics. It was found that the device parameters of the solar cells under study are unaffected until a certain level of defect concentration (1 × 10^13^ cm^–3^) is attained. At an *O*_*i*_ concentration of 1 × 10^14^ cm^–3^, a slight increase in the efficiency can be observed in doped devices. However, the observed increase was not exhibited by the CO device. A further increase in the *O*_*i*_ concentration decreased the efficiency, *J*_sc_, and *V*_oc_ of all the devices. The variation in FF showed a slightly different trend compared to other parameters. The FF decreased at a higher defect concentration in the case of the CO device, whereas for all the doped devices, the FF showed an increase at a higher doping level of defect density.

**Figure 7:**
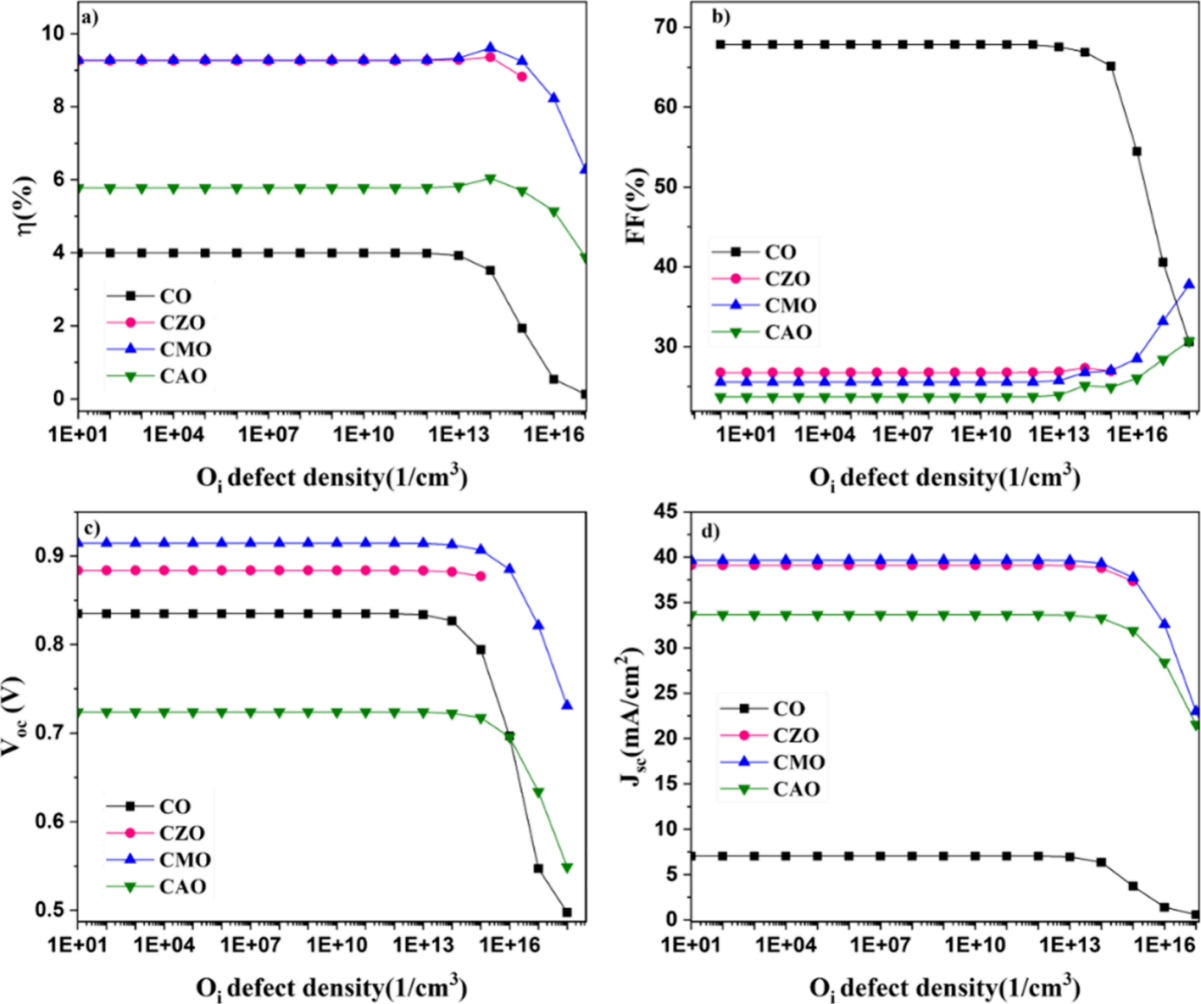
Influence of *O_i_* defect concentration on the device parameters (a) efficiency, (b) fill factor, (c) open circuit voltage, and (d) current density of simulated solar cells.

The decline in efficiency is associated with the decrease in the *J*_sc_. Increasing defect density accelerates the recombination rate of photogenerated charge carriers, resulting in a decrease in *J*_sc_, *V*_oc_, and consequently, η.^22^ However, the unexpected improvement in the efficiency of the doped samples at an *O*_*i*_ defect level of 1 × 10^14^ cm^–3^ in the doped samples might be due to the enhanced conductivity of the absorber layer. Increasing the conductivity of the absorber layer reduces parasitic resistances, which, in turn, enhances FF and thus improves the efficiency of the devices. This improvement is attributed to the reduction in resistive losses within the solar cell structure, leading to a better overall performance. As the doping concentration increases, *R*_s_ decreases leading to the increase in FF at higher *O*_*i*_ defect concentration. However, as the defect concentration increases, the scattering of the charge carriers can also increase, thereby decreasing the *J*_sc_ and efficiency in doped samples.^23^ Thus, the CuO solar cell performance can be improved by introducing a controlled level of *O_i_* defects.

The performance of solar cells was examined to understand the effects of *V*_*o*_ defects, specifically by introducing a defect level located 0.69 eV above the VB.^21^ The defect concentration was varied from 0 to 1× 10^18^ cm^–3^ and the observed change in the device performance is shown in Figure 8. The presence of *V*_*o*_ defects did not impact the operational characteristics of solar cell devices initially. Up to a defect concentration of 1× 10^15^ cm^–3^, the device parameters remained relatively stable. However, beyond this concentration, there was a noticeable decline in the device performance. It is noteworthy that the carrier concentration of the absorber layer used in the simulations was 1× 10^16^ cm^–3^. Therefore, as the concentration of *V*_*o*_ defects exceeds the carrier density, the performance of the solar cells decreases, which aligns with expected behavior in such scenarios. As the *V*_*o*_ defects increase, being a donor defect, they decrease the p-type conductivity of the CuO absorber layer, thereby leading to a decrease in *J*_sc_ and hence the efficiency. It can be observed that the influence of *V*_*o*_ defects is greater on *J*_sc_ compared to *V*_oc_ as there is a sharp decline in *J*_sc_ at higher concentrations whereas the reduction is *V*_oc_ is comparatively lower. As observed for the *O*_*i*_ defects, the efficiency and FF of doped devices show a small increase at 1× 10^14^ cm^–3^. This suggests that when defect density is less than this level, it hardly affects the performance of CuO-based solar cells.

**Figure 8:**
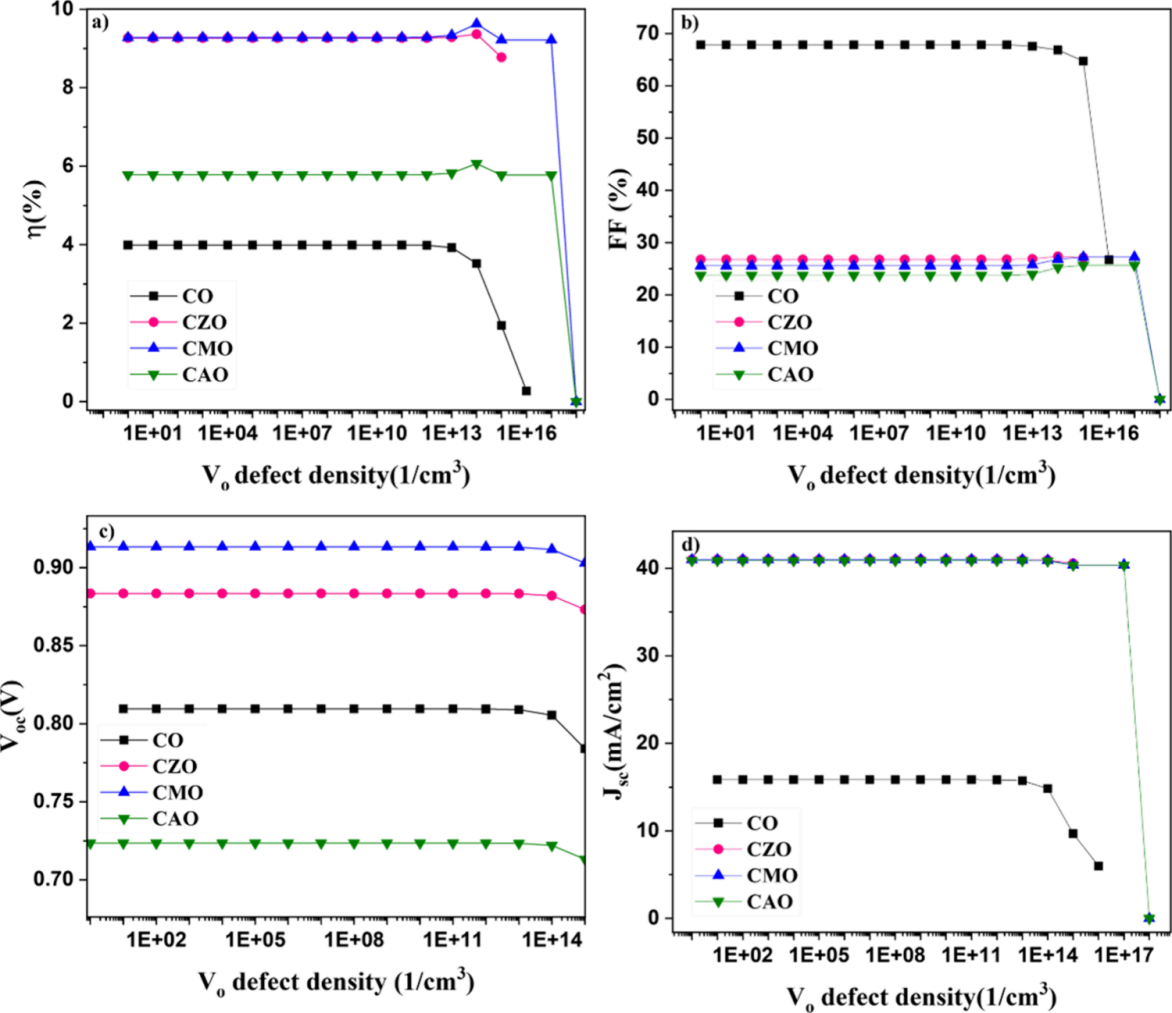
Influence of *V_o_* defect concentration on the device parameters (a) efficiency, (b) fill factor, (c) open circuit voltage, and (d) current density of simulated solar cells.

In a real system, both *O*_*i*_ and *V*_*o*_ defects coexist and therefore it is important to study the combined effect of *O*_*i*_ and *V*_*o*_ defects. This was understood by varying both the *O*_*i*_ and *V*_*o*_ defects between 1× 10^10^ and 1× 10^15^ cm^–3^ and Figure 9 depicts the variation in efficiency. For the CO sample, the highest efficiency of 3.99% was observed when both defect concentrations were kept at the minimum level. Even though the efficiency decreased with the increase in defect density, an interesting observation was that the device efficiency was higher when *O*_*i*_ defects dominated over *V*_*o*_ defects. However, a very large increase in the *O*_*i*_ defects has negatively influenced the efficiency as a higher amount of *O*_*i*_ can result in charge scattering.

**Figure 9:**
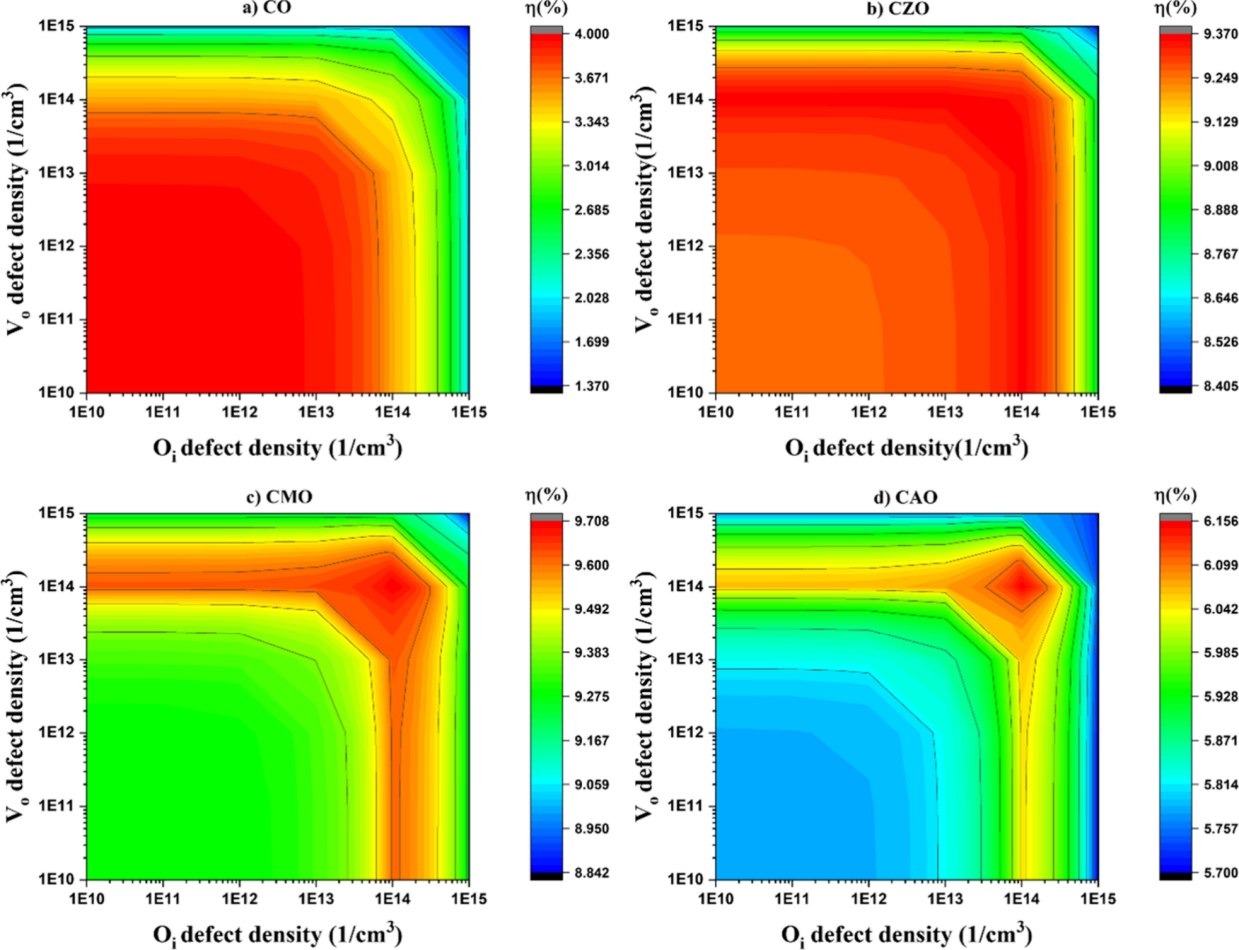
Influence of both *V_o_* and *O_i_* defect concentration on the performance of CuO solar cells.

Doping has, however, increased the tolerance of defect density in CuO solar cells. After doping, the highest efficiency is observed at a defect density of 1× 10^14^ cm^–3^. The efficiency of the doped devices was higher when the *O*_*i*_ defects dominated the *V*_*o*_ defects. An improvement in efficiency was attained when *O*_*i*_ defect density was 10 times that of *V*_*o*_ defect density. The observed enhancement in the efficiency is due to the improvement in the p-type conductivity due to the *O*_*i*_ defects. When *V*_*o*_ defects dominate the *O*_*i*_ defects, p-type conductivity of the CuO decreases due to the extra electrons from the *V*_*o*_ defects. This observation is crucial as in the experimental studies of the doped samples, suggesting an enhancement of *O*_*i*_ defects after doping. This interesting observation suggests that controlling the oxygen defects can enhance the efficiency of CuO solar cells.

### 3.2 Fabrication and Characterizations

#### Structural Characterization

GI-XRD was used to understand the structural properties of the devices. GI-XRD of all the devices were taken for three different incident angles, 0.3, 0.5, and 1°. The obtained diffractograms for all of the devices are given in Figure 10. All the films showed consistent diffraction patterns corresponding to the monoclinic phase of CuO. All the observed peaks were identified and matched with the standard JCPDS card (01–077–7718), which confirmed the crystal structure of the films. No other peaks corresponding to any secondary phases of copper oxide or dopant oxides were observed, which exhibits the phase purity of the samples. This indicates that dopant ions were well substituted into the Cu^2+^ lattice sites without affecting the CuO crystal structure. The diffractograms of the films showed two major peaks at ∼35.5 ° and ∼38.8 °. These peaks correspond to (1̅11) and (111) reflection planes. In addition to these two peaks, several minor peaks of monoclinic CuO were also identified. As compared to the films on glass substrates, the peak positions were observed to shift toward a lower angle. This shift in the peak position may be due to the different growth rates in different substrates. Also, as the films were deposited at higher temperature, the molecules of the films gained higher energy, leading to lattice expansion.

**Figure 10:**
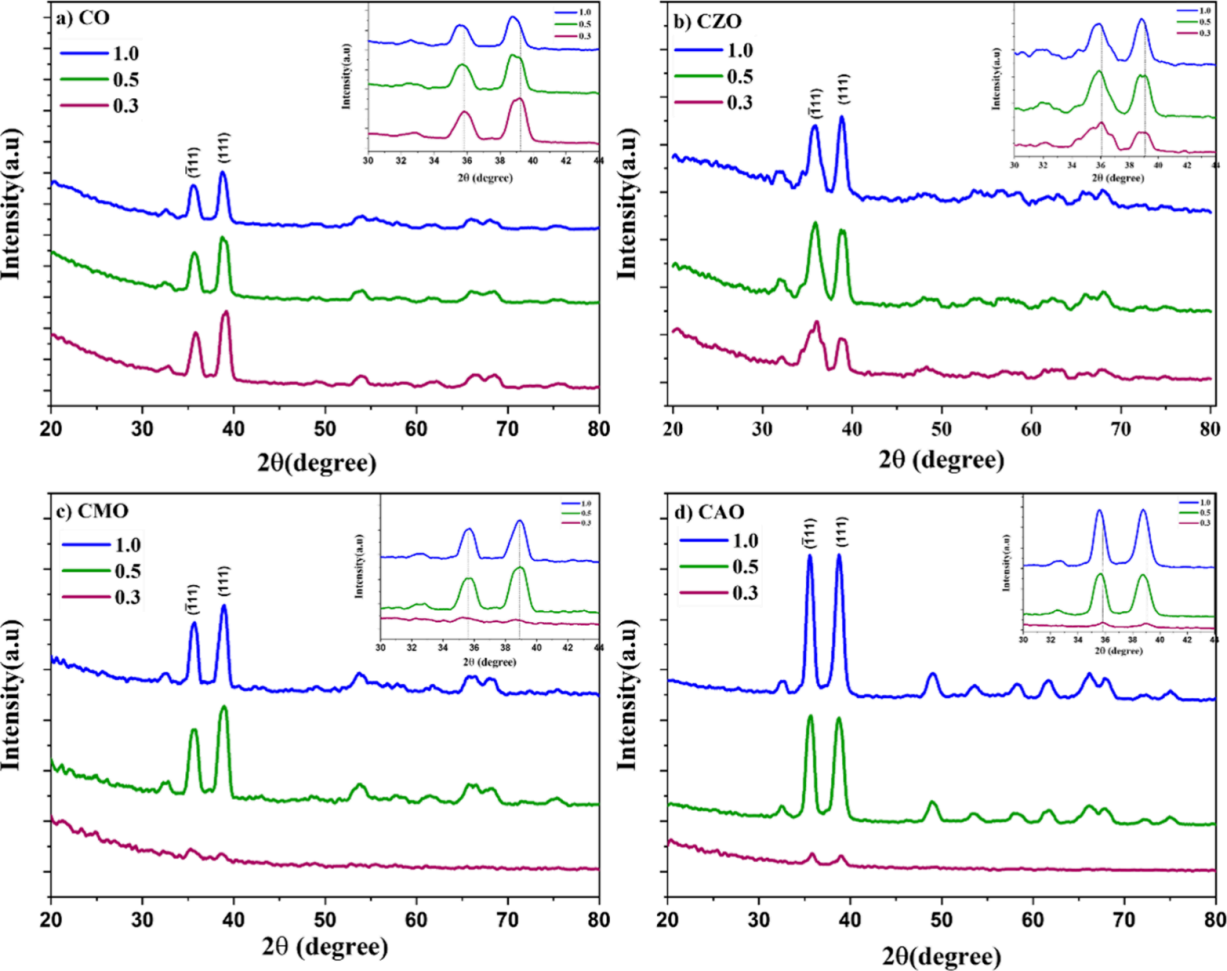
GI-XRD of fabricated Si heterostructures (inset shows the shift in the peak positions of the two major peaks (1̅11) and (111) with the incidence angle).

In the inset of Figure 10, the shift in the peak position of the films at various incident angles is depicted. It was noted that with increasing incident angle, the XRD peaks shifted toward lower angles for all films except CMO. For CMO, the XRD peaks showed a nominal shift toward the right. According to the growth mechanism, thin films are formed in layers and during each deposition, atoms of the layers occupy the minimum potential energy. The growth and orientation of these films are significantly influenced by the substrate’s orientation. Si has a lattice parameter of 5.4 Å, whereas the lattice parameters of CuO are *a* = 4.6 Å, *b* = 3.4 Å, and *c* = 5.1 Å. Hence, as we move deeper into the films, we can observe that the films are trying to align more and more with the substrate lattice leading to the shift in peak position. This indicates that CuO lattice is trying to expand resulting in the shift of the peak toward lower angles. The nominal right shift in CMO is due to the smaller ionic radii of Mg^2+^ compared to those of the Cu^2+^ ions.

The intensity of the diffraction peaks notably varied with changes in the incident angle. With an increase in the incident angle, reflection from a greater number of layers is obtained, and hence, the obtained diffraction pattern has a higher intensity for deeper penetration. A comparison of the intensity of diffraction peaks at the highest incident angle shows that the maximum intensity was observed for CAO followed by that for CO, CMO, and CZO having nearly similar intensities. The crystallinity of the sample can be associated with peak intensity. A higher intensity indicates better crystallinity.

#### Morphological Characterization

The FESEM images of the prepared samples (20 kx, scale bar 1 μm) are shown in Figure 11. All the films displayed high uniformity across the sample surface, with no visible variations or nonuniform growth. Furthermore, there were no indications of cracks or discontinuities in the films, indicating their high quality. Examination of the micrographs revealed a consistent surface morphology among the films. Except for the CZO sample, all others showed a similar morphology. Close observation of the CZO sample showed rod-like growth of the nanoparticles. The FESEM micrographs revealed that the CAO film exhibited the largest grain size compared to that of the other samples. Despite variations in substrates, the morphology of all samples closely resembled that observed in films grown on glass substrates. This suggests that the choice of substrate did not significantly affect the morphology of the samples.

**Figure 11:**
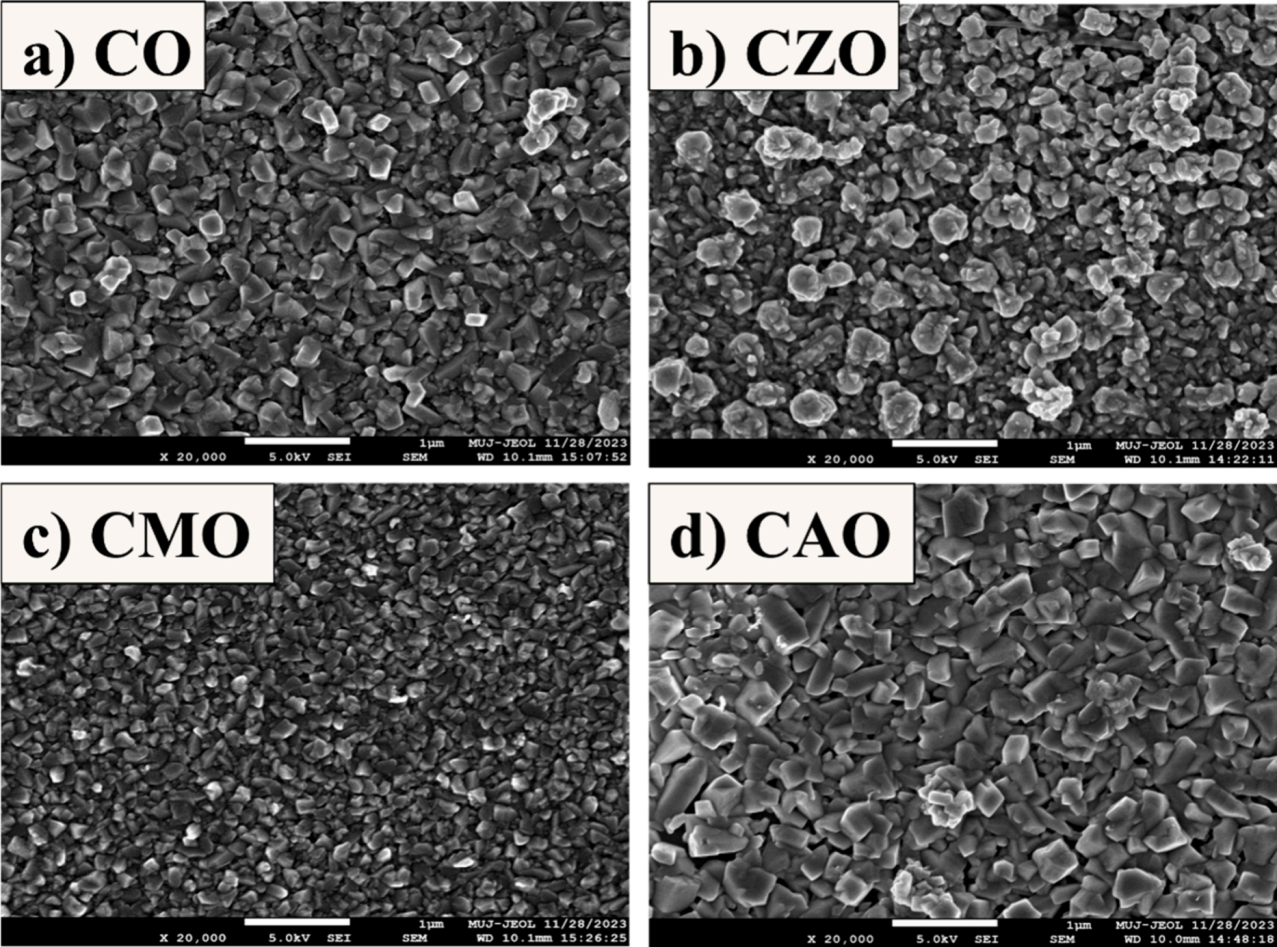
Surface micrographs of (a) CO, (b) CZO, (c) CMO, and (d) CAO.

#### Optical Characterization

Figure 12 illustrates the reflectance spectra of the prepared heterostructures. All of the films showed low reflectance in the visible wavelengths. This indicates the suitability of these devices for solar cell applications. Doping has decreased the reflectance of the films between a wavelength range of 500–1000 nm. Among the four devices, CZO showed the least reflectance. This low value of reflectance can be due to the rod-like morphology of the films that results in a much rougher surface as compared to other films, thereby improving the absorption. Low values of the reflectance suggest that the fabricated devices have good optical properties suitable for solar cell applications.

**Figure 12:**
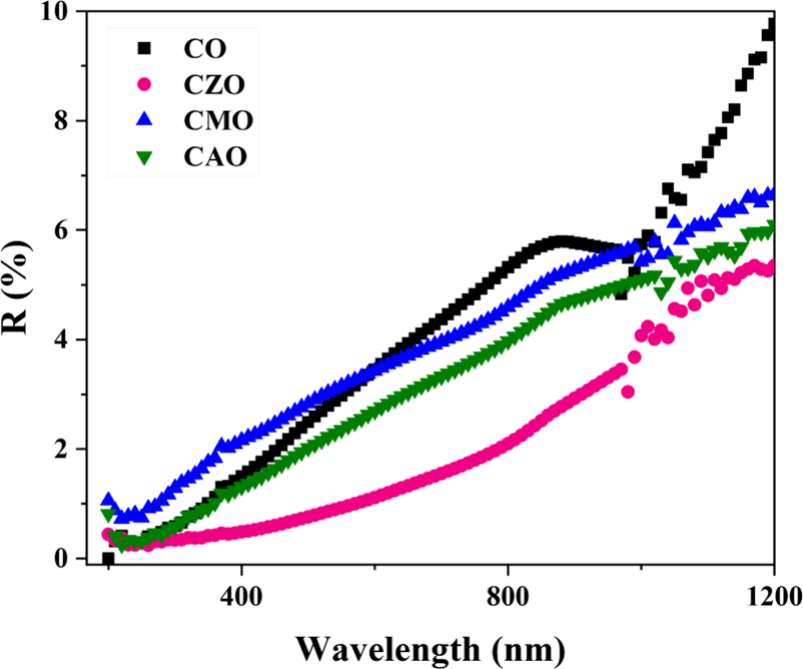
Reflectance spectra of the fabricated devices.

The band gap of the deposited thin films was obtained from reflectance spectra using Kubelka–Munk function *F*(*R*_∞_) given by

$$F\left(R_{\infty }\right)=\frac{K}{S}=\frac{{\left(1-R_{\infty }\right)}^{2}}{2R_{\infty }}$$ 6

where *R*_∞_ is the reflectance of an infinitely thick sample, *S* is the scattering coefficient, and *K* is the absorption coefficient. Substituting *F*(*R*_∞_) in Tauc’s relation

$$\alpha h\nu =B{\left(h\nu -E_{g}\right)}^{n}$$ 7

we have

$$F\left(R_{\infty }\right)h\nu =B{\left(h\nu -E_{g}\right)}^{n}$$ 8

The direct band gap is then estimated from the *x*-intercept of *h*ν vs (*F*(*R*_∞_)*h*ν)^2^. The estimated band gaps of CO, CZO, CMO, and CAO samples are 1.50, 1.54, 1.57, and 1.38 eV, respectively.

#### Raman Analysis

The Raman spectra of the fabricated heterostructures are presented in Figure 13. Each sample exhibits four distinct Raman active modes centered at approximately 302, 352, 525, and 634 cm^–1^. The peaks at 302, 352, and 634 cm^–1^ are associated with A_g_, B_g_^1^, and B_g_^2^ modes, respectively, which align well with values reported in the existing literature. Additionally, the Raman spectra feature a prominent peak at 525 cm^–1^, which is attributable to the Raman signature of the Si substrate employed. Beyond these main peaks, the spectra also reveal a broad peak at 1110 cm^–1^, indicative of the multiphonon mode.

**Figure 13:**
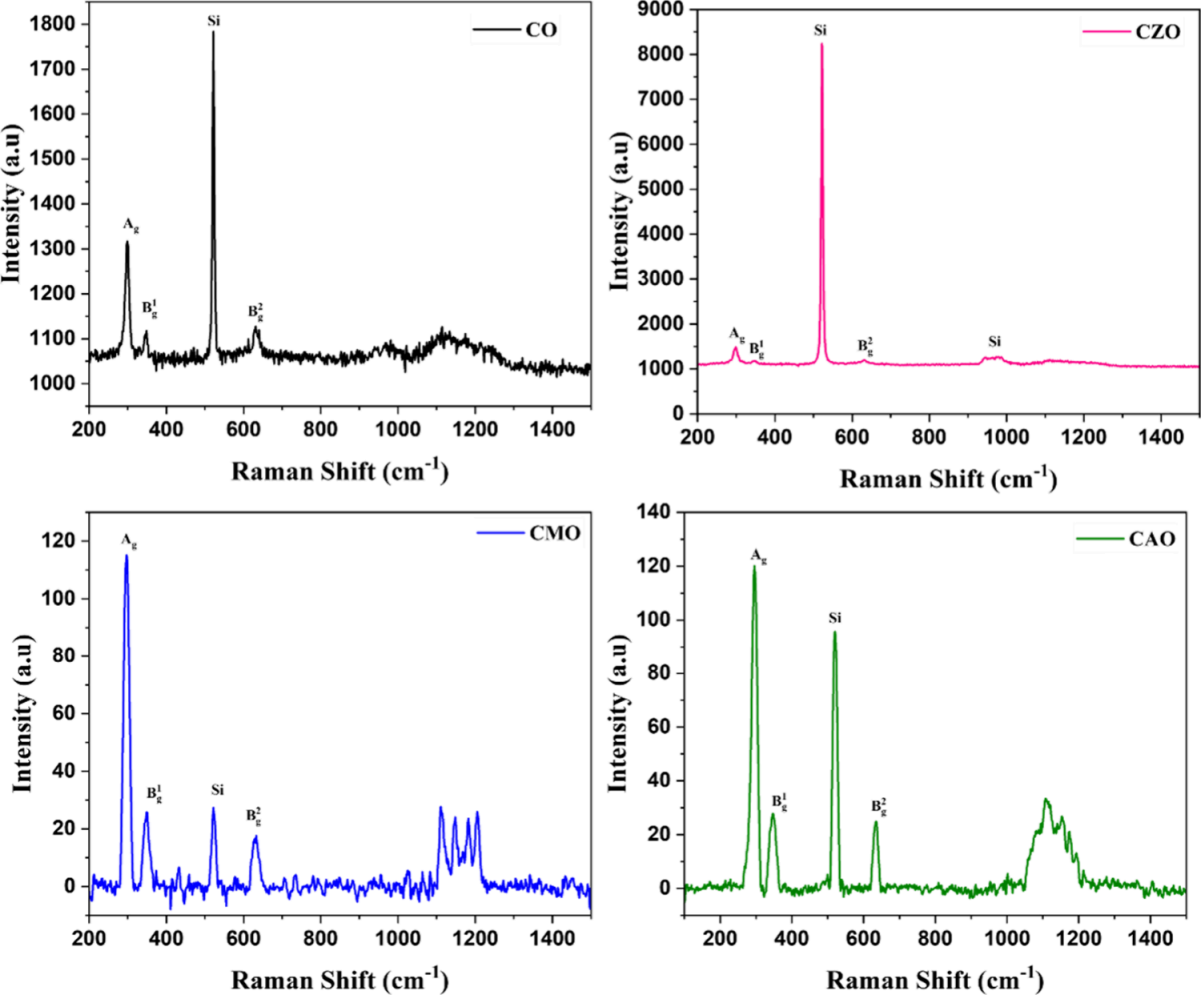
Raman spectra of fabricated Si-heterostructures.

The Raman spectra of the devices revealed a shift toward longer wavelengths in their peak positions compared to films grown on glass substrates, indicating a red shift. This shift is indicative of the presence of tensile stress within the films, a correlation that aligns with the observations made using XRD. The intensity variations are attributed to differences in the growth mechanisms of the films on Si and glass substrates, highlighting the influence of the substrate material on the structural properties of the films. Also, the dopant can affect the peak intensity.

The chemical homogeneity of the prepared films is studied through Raman mapping. The Raman mapping of the A_g_ peak in the sample is shown in Figure 14. The uniformity in the distribution of the color indicates the chemical homogeneity of the films. Among the four samples, the highest uniformity is observed in CAO films for which the color is mostly uniformly distributed throughout. This indicates the good quality of CAO films. The obtained result can also be compared with device performance, which is discussed in the next section.

**Figure 14:**
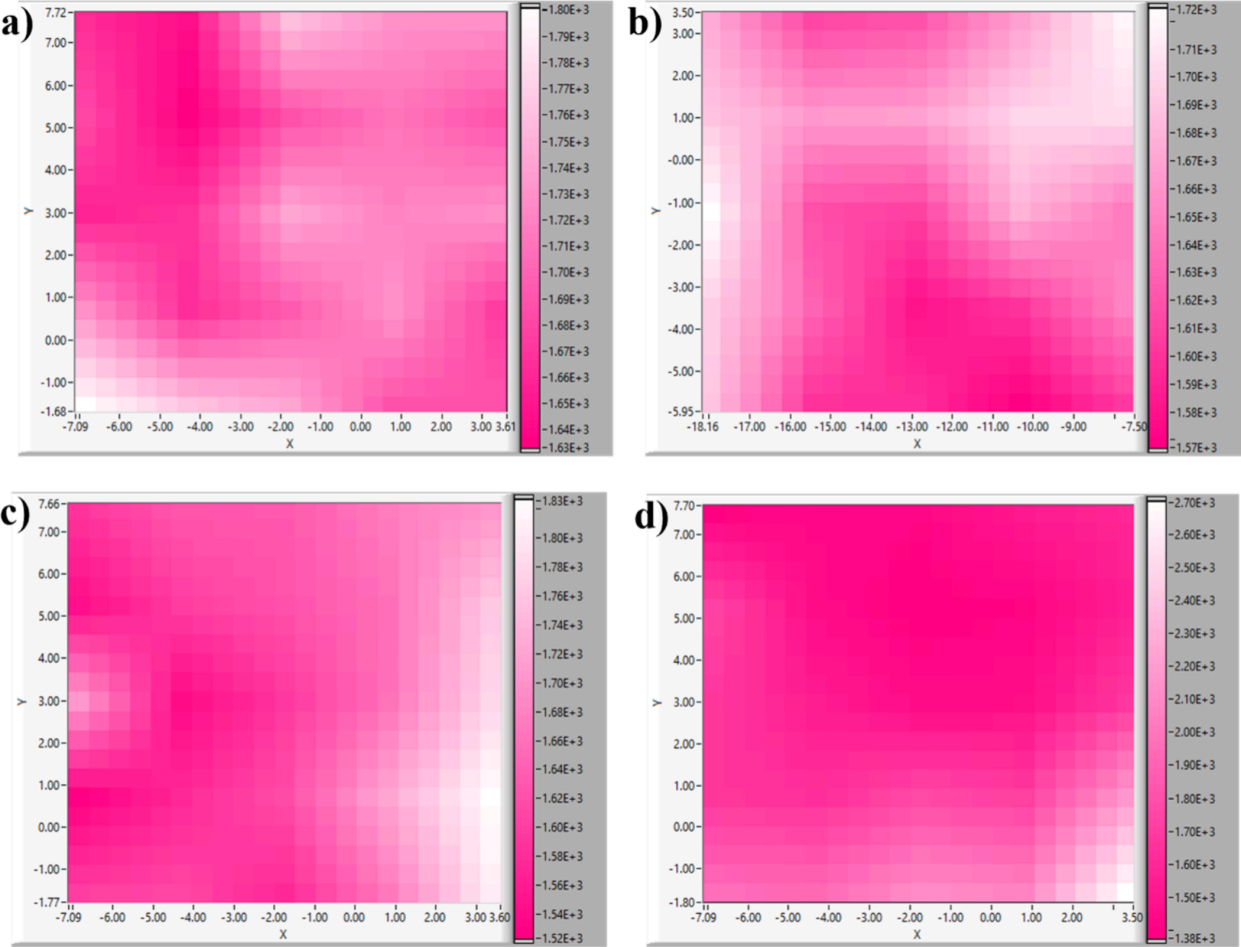
Raman mapping of A_g_ peak in (a) CO, (b) CZO, (c) CMO, and (d) CAO devices.

#### *I*–*V* Analysis

The electrical characteristics and photovoltaic properties were analyzed from dark and illumination *I*–*V* measurements of the fabricated devices. The typical semilog *J*–*V* characteristics of the devices under dark and illumination are shown in Figure 15. All the samples showed rectifying behavior that confirms the formation of p–n junction. The diode parameters of the devices were calculated from the dark and illumination conditions using the Cheung model and the obtained values of the different parameters such as *n*, *R*_s_, and ϕ_*b*_ are given Table 4. The *n* value measures how close the behavior of the fabricated heterojunction is to the ideal diode behavior. The value of *n* provides information about charge transport as well as recombination. For an ideal diode, *n* = 1, implies that only diffusion current, corresponding to band-to-band recombination, flows through the p–n junction. When *n* = 2, the device current is dominated by the generation and recombination of charge carriers, which involves the defect or trap states within the interface gap.^24^

**Table 4:** Diode Parameters of Si Heterostructures

	**dark**			**illumination**		
**devices**	***n***	**ϕ**_*b*_ **(eV)**	***R***_**s**_ **(kΩ)**	***n***	**ϕ**_*b*_ **(eV)**	***R***_**s**_ **(kΩ)**
**CO**	1.30	1.12	304	2.52	0.66	197
**CZO**	2.20	0.88	9	1.33	0.88	128
**CMO**	2.12	0.80	2	1.27	0.99	506
**CAO**	2.12	0.86	34	2.71	0.47	376

**Figure 15:**
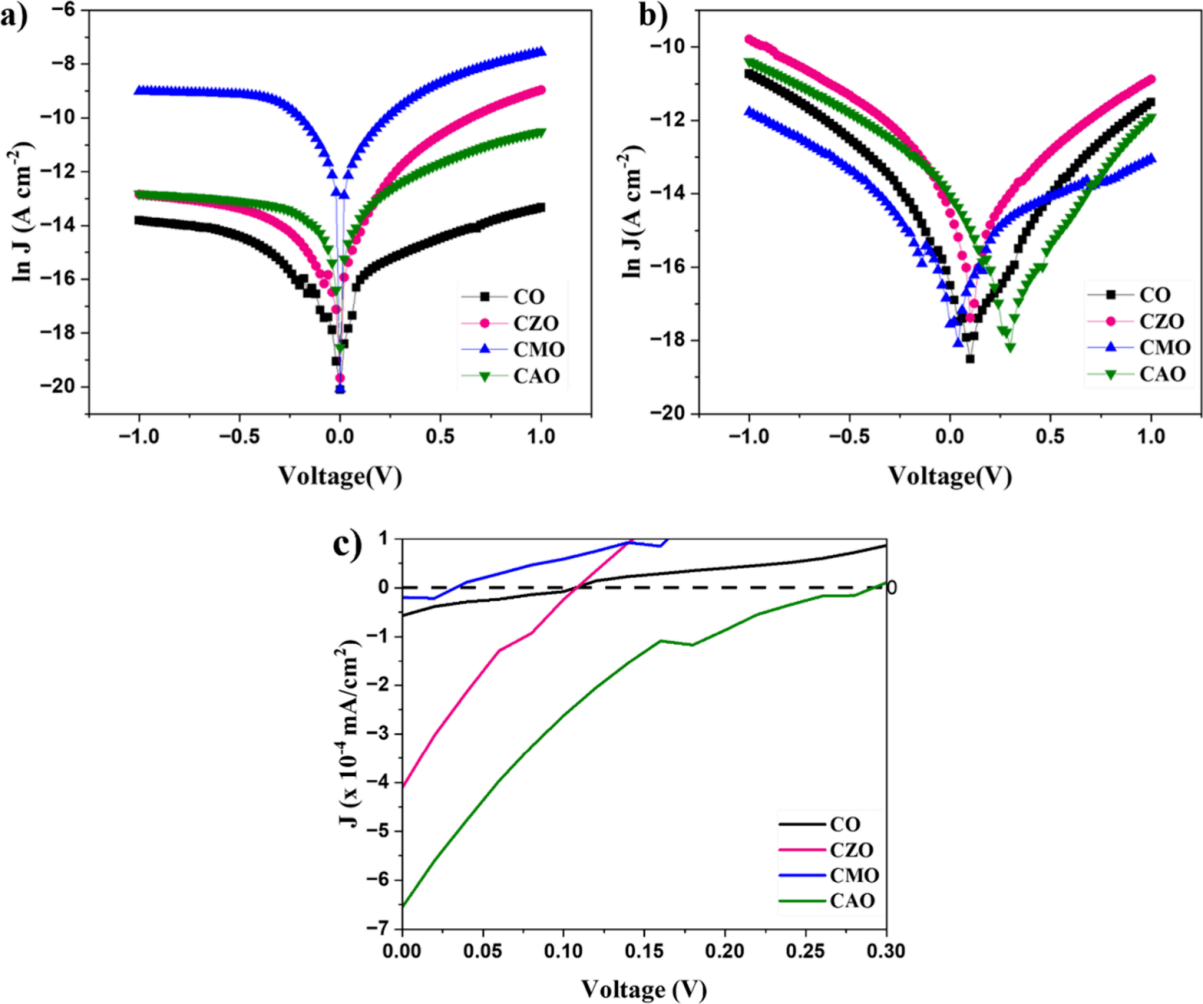
Plots of ln *J* vs V characteristics of Si-heterostructures under (a) dark and (b) illumination conditions, (c) zoomed-in view of *J* vs *V* under illumination.

The ideality factor of the devices was found to be 1.3 for CO and greater than 2 for all the doped devices having doped CuO as p-type layer. This deviation from the ideal behavior is expected after doping because doping introduces defect states that act as traps distributed across the interface and surface, leading to recombination and thereby increasing the *n* value.^25^ In addition to these, the presence of an inhomogeneous interface layer can also lead to an increase in *n* values.^25^ Further, the high leakage current of the samples indicates the existence of nonradiative recombination and interface defects. The barrier height showed a decreasing trend after doping. The decrease in the barrier height may be due to the modification in the energy levels after doping.^25^ A significant reduction in the series resistance after doping is due to the increased conductivity of the absorber layer.

Ultraviolet photoelectron spectroscopy (UPS) was employed to determine the work function of the individual layers of all four heterostructures. The work function of Ag is taken as 4.65 eV.^26^ The work function was calculated by subtracting the secondary cut off energy from the incident energy (40 eV) and the obtained values are 6.13, 5.48, 5.82, and 5.34 eV for CO, CZO, CMO, and CAO, respectively. From the upper and lower onset energies for individual layers, the important parameters that are required to draw the band diagram have been calculated and the band diagram of CAO heterostructure is shown in Figure 1(b). It is observed that there is a shift in the position of the Fermi level toward the VB after doping. This indicates that p-type conductivity is indeed enhanced after doping. In addition to this, the obtained barrier height from UPS measurement follows similar trend as observed in the *I*–*V* measurements. The UPS spectra of the absorber layer of the heterostructures are given in Figure 16.

**Figure 16:**
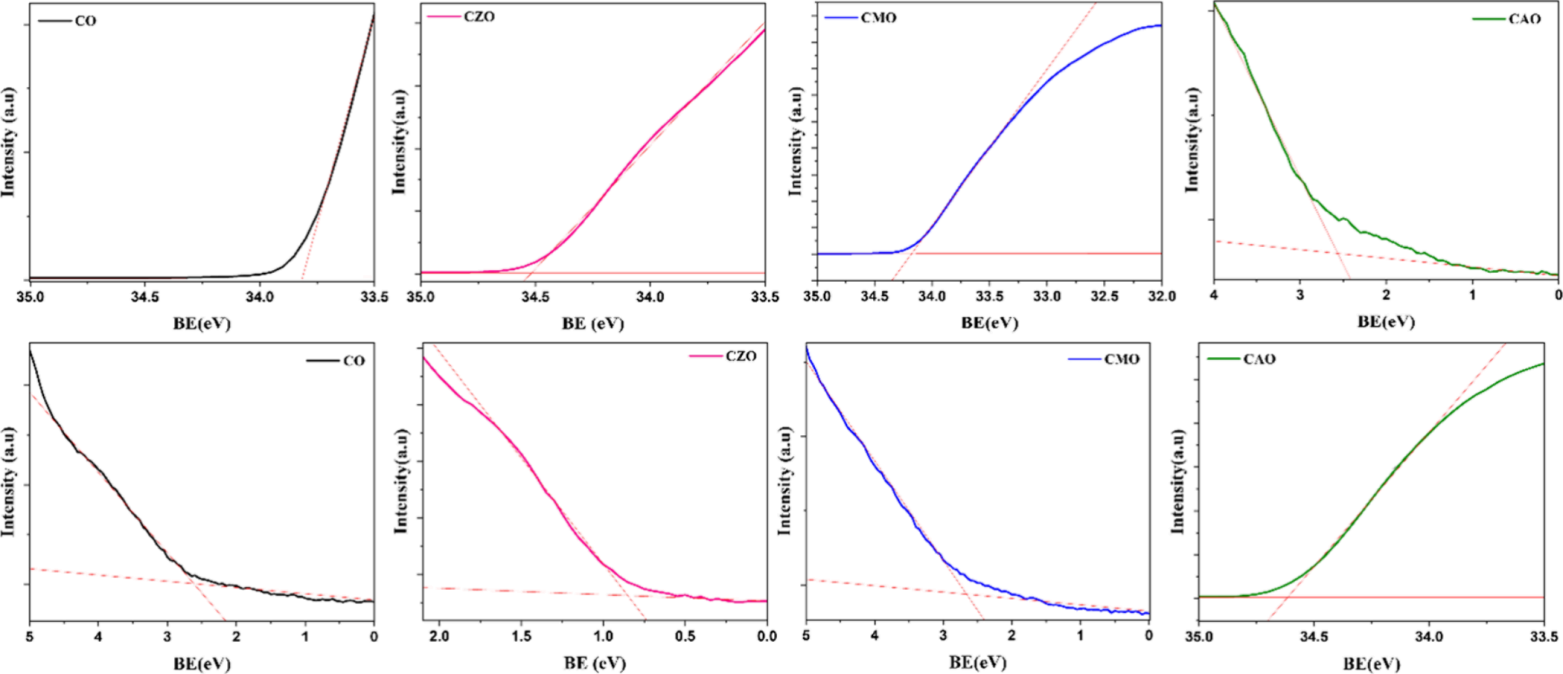
UPS spectrograph of the absorber layer of CO, CZO, CMO, and CAO devices.

After illumination, the CZO and CMO samples showed a decrease in the ideality factor and an increase in the barrier height, whereas the CO and CAO samples exhibited an increase in the ideality factor and a decrease in the barrier height. Illumination generates additional charge carriers, causing deviations from ideal diode behavior and thus an increase in the ideality factor. Conversely, the decrease in the ideality factor after illumination is attributed to the presence of an inhomogeneous interface layer. This layer consists of regions with varying barrier heights, where in dark conditions, electrons can traverse regions with lower barriers dominating current flow, resulting in a large ideality factor.^27^ Under illumination, more electrons gain sufficient energy to traverse regions with higher barriers, leading to an increase in barrier height and a lower ideality factor thereafter.^27^ The series resistance is observed to be increased after illumination due to the increase in the lateral flow of electrons in the emitter.^28^

The zoomed-in view of the fourth quadrant of *I*–*V* under solar illumination is shown in Figure 15(c). All the devices showed photovoltaic properties. A significant improvement in *J*_sc_ was observed with Al and Zn doping. However, Mg doping reduced the *J*_sc_ values. The highest *V*_oc_ of 0.29 V was exhibited by CAO. While CZO and CO samples had the same Voc, the *V*_oc_ of the Mg-doped sample had decreased significantly. Therefore, the performance of CuO solar cells was significantly enhanced by improving their structural, optical, and electrical properties after doping. However, among the three doped devices, the CMO sample exhibited a very low photovoltaic response, indicating unsuitability for solar cell applications. The poor performance of Mg-doped samples can be attributed to inferior structural and optical properties of the films, resulting in overall reduced solar cell performance. Consequently, further parameter extraction for Mg-doped devices was deemed unnecessary.

When light is incident on a solar cell, only the photons that reach the junction contribute to the cell’s performance, while a portion is reflected. Therefore, internal efficiency is crucial for studying the behavior of the device. Al doping significantly enhanced the efficiency of the solar cells by nearly 15 times. For instance, the efficiency of CO increased from 0.42% to 6.52% after Al doping, showcasing a substantial improvement. Zn doping also resulted in a nominal efficiency increase to 0.91%. Despite these enhancements, the solar cells still exhibit low efficiency compared to its contenders. This inefficiency primarily stems from parasitic resistances, which notably reduce the FF of the devices. The shape of the *I*–*V* curve, influenced by FF, appears convex due to the high barrier height between the absorber and metal electrode, causing the accumulation of charge carriers and inefficient dissociation of excitons.^24^

The experimental results thereby validated the numerical simulation results. However, the obtained trend is not the same as that predicted from the simulations. According to the simulations, CMO was expected to exhibit the highest efficiency, while CAO was anticipated to show the lowest. However, the experimental results revealed the opposite trend. This discrepancy can be attributed to limitations in the numerical calculations, which accounted for only the optical properties of the films and did not consider their structural characteristics. In reality, the structural properties of films significantly influence their electrical properties and, hence, impact the performance of solar cells. This oversight likely contributed to the deviation of the experimental results.

Among the devices, CAO films exhibited superior structural, optical, and electrical properties compared with CMO and CZO films. Examination of the FESEM images indicated that CMO films had the smallest grains among all films. Such small grains can lead to increased defects, which, in turn, can facilitate the recombination of charge carriers, thereby explaining the poor performance of CMO films. Additionally, the quality of the junction also plays a crucial role in determining the properties of solar cells, a factor that was not addressed in the numerical simulations. The device parameters of the fabricated solar cells are detailed in Table 5.

**Table 5:** Device Parameters of the Fabricated Heterojunction Solar Cells

**device**	*V*_**oc**_ **(V)**	*J*_**sc**_ **(×10** ^**–5**^ **mA cm** ^**–2**^ **)**	**FF (%)**	**η (%)**
**CO**	0.11	6.84	22	0.42
**CZO**	0.11	49.17	19	0.91
**CMO**	0.03	2.37		
**CAO**	0.29	78.37	14	6.52

## 4 Conclusions

SCAPS simulations were employed to evaluate the suitability of both undoped and doped CuO thin films as absorbers for solar cells. The study comprehensively examined the impact of the absorber layer thickness, doping, and presence of oxygen defects on solar cell performance. The thickness optimization study showed that the optimum thickness for CO, CZO, CMO, and CAO absorber layers were 16, 5, 5.5, and 5 μm, respectively. Doping has significantly improved the performance of CuO solar cells from about 5% in the CO device to about 31%, 32%, and 25% in CZO, CMO, and CAO devices, respectively. Enhanced optical properties and band gap tuning resulting from absorber layer doping were linked to the observed performance improvements. Doping has also helped in reducing the absorber layer thickness, which helps in cost reduction of the CuO solar cells. The obtained device parameters are also influenced by parasitic resistances. The FF of the devices reduce significantly with the introduction of *R*_s_ and *R*_sh_. The intrinsic defects within the absorber layer significantly influence the solar cell performance. Therefore, the impact of oxygen defects, *V*_*o*_ and *O*_*i*_, on the device parameters was studied. It was observed that the efficiency of the devices can be improved when *O*_*i*_ defects dominate over *V*_*o*_ defects. Doping has also enhanced the defect tolerance in CuO devices. Therefore, the simulation study offers insights into the parameters that should be adjusted in the CuO absorber layer during fabrication to achieve enhanced performance in CuO/Si solar cells.

Si heterostructures incorporating CuO and doped CuO absorber layers were fabricated by using the spray pyrolysis technique, and their properties were thoroughly investigated by using various characterization methods. Doping significantly influenced the photovoltaic properties of the films, with Zn and Al dopants enhancing cell performance, while Mg doping had a detrimental effect. The efficiency of Si/CuO solar cells increased after doping, rising from 0.42% in undoped CuO to 6.52% in 1 at. % Al doped CuO solar cells. This notable efficiency enhancement can be attributed to improvements in the structural, optical, and electrical properties of the films. Importantly, experimental results corroborated the findings from simulations, underscoring the suitability of undoped and doped CuO absorber layers deposited via spray pyrolysis for sustainable and cost-effective solar cell applications.

## References

[1] Shen Y., Guo M., Xia X., Shao G. (2015). Role of materials chemistry on the
electrical/electronic properties of CuO thin films. Acta Mater..

[2] Chowdhury T. A. (2023). Simulation
Study of CuO-Based Solar Cell with Different Buffer Layers Using SCAPS-1D. Energy Power Eng..

[3] Jahan N. (2025). A comparative study
of CuO based solar cell with ZnTe HTL and SnS2
ETL using SCAPS 1D simulation. Journal of Optics.

[4] Ahmmed S., Aktar A., Tabassum S., Rahman M. H., Rahman M. F., Md. Ismail A. B. (2021). CuO based
solar cell with V2O5 BSF layer: Theoretical
validation of experimental data. Superlattices
Microstruct..

[5] Benaissa N. (2023). Experimental and numerical
simulation studies of CuO thin films based
solar cells. Engineering Research Express.

[6] Gao F., Liu X. J., Zhang J. S., Song M. Z., Li N. (2012). Photovoltaic
properties of the p-CuO/n-Si heterojunction prepared through reactive
magnetron sputtering. J. Appl. Phys..

[7] Masudy-Panah S. (2015). p-CuO/n-Si
heterojunction solar cells with high open circuit voltage
and photocurrent through interfacial engineering. Progress in Photovoltaics: Research and Applications.

[8] Masudy-Panah S., Radhakrishnan K., Tan H. R., Yi R., Wong T. I., Dalapati G. K. (2015). Titanium
doped cupric oxide for photovoltaic application. Sol. Energy Mater. Sol. Cells.

[9] Masudy-Panah S., Radhakrishnan K., Kumar A., Wong T. I., Yi R., Dalapati G. K. (2015). Optical bandgap widening and phase transformation of
nitrogen doped cupric oxide. J. Appl. Phys..

[10] Prakash A., V S G. K., Moger S. N., Mahesha M. G. (2022). Spectroscopic and
electrical analysis of spray deposited copper oxide thin films. Mater. Today Commun..

[11] Prakash A., Mahesha M. G. (2023). Harnessing the tunability
of intrinsic defects in isovalent
Zn doped spray deposited CuO thin films. Mater.
Chem. Phys..

[12] Prakash A., Mishra V., Mahesha M. G. (2024). Development of enduring interstitial
defects in Mg-doped CuO thin films†. RSC Adv..

[13] Prakash A., Mishra V., Mahesha M. G. (2024). Probing intrinsic defects of aluminium-doped
CuO thin films for solar cell applications. RSC Adv..

[14] Burgelman M., Nollet P., Degrave S. (2000). Modelling polycrystalline semiconductor
solar cells. Thin Solid Films.

[15] Decock K., Zabierowski P., Burgelman M. (2012). Modeling metastabilities in chalcopyrite-based
thin film solar cells. J. Appl. Phys..

[16] Ait
Abdelkadir A., Oublal E., Sahal M., Gibaud A. (2022). Numerical
simulation and optimization of n-Al-ZnO/n-CdS/p-CZTSe/p-NiO (HTL)/Mo
solar cell system using SCAPS-1D. Results in
Optics.

[17] Ragb O., Mohamed M., Matbuly M. S., Civalek O. (2021). An accurate numerical
approach for studying perovskite solar cells. Int. J. Energy Res..

[18] Noman, M. A. A. ; Abden, M. J. ; Islam, M. A. Germanium Telluride Absorber Layer, A proposal for Low Illumination Photovoltaic Application Using AMPS 1D. In 2018 International Conference on Computer, Communication, Chemical, Material and Electronic Engineering (IC4ME2); IEEE, 2018; pp 1–5. 10.1109/IC4ME2.2018.8465494.

[19] Mortadi A., El Hafidi E., Monkade M., El Moznine R. (2024). Investigating
the influence of absorber layer thickness on the performance of perovskite
solar cells: A combined simulation and impedance spectroscopy study. Mater. Sci. Energy Technol..

[20] Solanki, C. S. Solar Photovoltaics: Fundamentals, Technologies and Applications; Phi learning pvt. Ltd. 2017.

[21] Živković A., de Leeuw N. H. (2020). Exploring the formation
of intrinsic p-type and n-type
defects in CuO. Phys. Rev. Mater..

[22] Samiul
Islam M. (2021). Defect Study and Modelling of SnX3-Based Perovskite
Solar Cells with SCAPS-1D. Nanomaterials.

[23] Paknazar, P. ; Shakiba, M. Role of Doping Concentration of n- and p-Strip Regions on Optoelectronical Characterization in IBC-SHJ Solar Cell. In 2023 5th Iranian International Conference on Microelectronics (IICM); IEEE, 2023; pp 36–40. 10.1109/IICM60532.2023.10443081.

[24] Muhammad F. F. (2017). Employment of single-diode
model to elucidate the variations in photovoltaic
parameters under different electrical and thermal conditions. PLoS One.

[25] Kaphle A., Echeverria E., McLlroy D. N., Hari P. (2020). Enhancement in the
performance of nanostructured CuO-ZnO solar cells by band alignment. RSC Adv..

[26] Keshav R., Mahesha M. G. (2021). Investigation on
performance of CdTe solar cells with
CdS and bilayer ZnS/CdS windows grown by thermal evaporation technique. Int. J. Energy Res..

[27] Kim S. H., Jung C. Y., Kim H., Cho Y., Kim D. W. (2015). Forward
current transport mechanism of Cu Schottky barrier formed on n-type
Ge Wafer. Transactions on Electrical and Electronic
Materials.

[28] Humada A. M., Hojabri M., Mekhilef S., Hamada H. M. (2016). Solar cell
parameters
extraction based on single and double-diode models: A review. Renewable and Sustainable Energy Reviews.

